# Seabird distribution patterns observed with fishing vessel’s radar reveal previously undescribed sub-meso-scale clusters

**DOI:** 10.1038/s41598-017-07480-6

**Published:** 2017-08-04

**Authors:** Camille Assali, Nicolas Bez, Yann Tremblay

**Affiliations:** 1 Université de Montpellier, UMR MARBEC 248: Marine Biodiversity, Exploitation and Conservation, Bat. 24 – CC 093 Place Eugène Bataillon, 34095 Montpellier cedex 5, France; 2 Institut de Recherche pour le Développement, UMR MARBEC 248: Marine Biodiversity, Exploitation and Conservation, Avenue Jean Monnet CS 30171, 34203 Sète cedex, France

## Abstract

Seabirds are known to concentrate on prey patches or at predators aggregations standing for potential feeding opportunities. They may search for prey using olfaction or by detecting visually feeding con-specifics and sub-surface predators, or even boats. Thus, they might form a foraging network. We hypothesized that conditionally to the existence of a foraging network, the visual detection ability of seabirds should have a bearing on their medium-scale distribution at sea. Using a fishing-boat radar to catch the instantaneous distribution of seabirds groups within 30 km around the vessel, we conducted a spatial clustering of the seabird-echoes. We found 7,657 clusters (i.e. aggregations of echoes), lasting less than 15 minutes and measuring 9.2 km in maximum length (median). Distances between seabirds groups within clusters showed little variation (median: 2.1 km; CV: 0.5), while area varied largely (median: 21.9 km^2^; CV: 0.8). Given existing data on seabirds’ reaction distances to boats or other marine predators, we suggest that these structures may represent active foraging sequences of seabirds spreading themselves in space such as to possibly cue on each others. These seabird clusters were not previously described and are size compatible with the existence of a foraging network.

## Introduction

Predators’ foraging strategies, such as seabirds’, have evolved so as to optimize prey searching^1, 2^, as well as prey capture rate^3–5^ in various prey availabilities and environmental conditions. Seabirds are known to concentrate on prey patches^6, 7^ or at foraging predators aggregations^8^, both standing for feeding opportunities^9^. When prey is not temporally or spatially predictable, seabirds may search for prey using olfactory cues^10^ or indirectly find prey patches by visually detecting other predators feeding and then decide to join the feeding aggregation^9^. Associations of marine predators (marine mammals, predatory fishes, seabirds) have been observed worldwide (eastern tropical Pacific^11, 12^; eastern central Atlantic^13^; south-western Indian^14^; tropical western Indian^15^). In tropical areas, where prey is scarce and heterogeneously distributed^16^, seabirds associations with tuna or dolphin are common and might be the main access to prey for many species of piscivorous seabirds^14, 17^.

Seabirds are known to aggregate rapidly on a patch of food^18, 19^ or when sub-surface predators demonstrate an active feeding behaviour^13^. This local enhancement may be more efficient with relatively high densities of seabirds^20, 21^. It supposes that a rapid information transfer exists among individuals who take into account other foragers’ behaviours to detect prey. This process may shape their spatial distribution within a foraging network^22^. Indeed, con-specifics and hetero-specifics have been shown to be important visual cues for prey location^14, 23, 24^. This implies that cues are within visual range. In theory, i.e. when the unique limitation is the line-of-sight range to the ocean horizon, seabirds’ ability to detect any visual cue is evaluated to max out at 20–50 km^8, 19^, depending on the flight height of the focal species. In Cape gannets, small groups of con-specifics have been proven to trigger some reactions by a forager at distances from a few hundreds meters up to 10 km^8^. Red-footed boobies flight recordings showed they regularly climbed at altitudes of 20–50 m, most likely to spot both prey movements at the surface and other seabirds behaviour^25^. The need for seabirds to maintain visual contact to take advantage of such a foraging network should induce an inter-individual optimal distance compromising between being too close, which reduces the searching range, and being too far, which breaks down the network. Seabirds distribution is then supposed to display an aggregation pattern which spatial structure would be compatible with individuals’ (or groups of individuals’) ability to cue on each other. From ship-transects observations, aggregative patterns in seabirds distribution have been detected and their chord length estimated to ~4.7–23 km^26^. However, to our knowledge, such aggregations have never been described in two spatial dimensions, neither over time. This is probably due to the difficulty in observing aggregations at this scale.

Marine radars, as the ones devoted and tuned for seabirds observation on board tuna purse-seiners, allow for an instantaneous recording of seabirds distribution at various ranges depending on radar types and power^27, 28^. Using this opportunity, the goals of this work were (1) to describe instantaneous spatial distributions of seabirds over time and (2) to verify whether they distribute themselves consistently with the use of information transfer between them.

## Results

**Figure 1:**
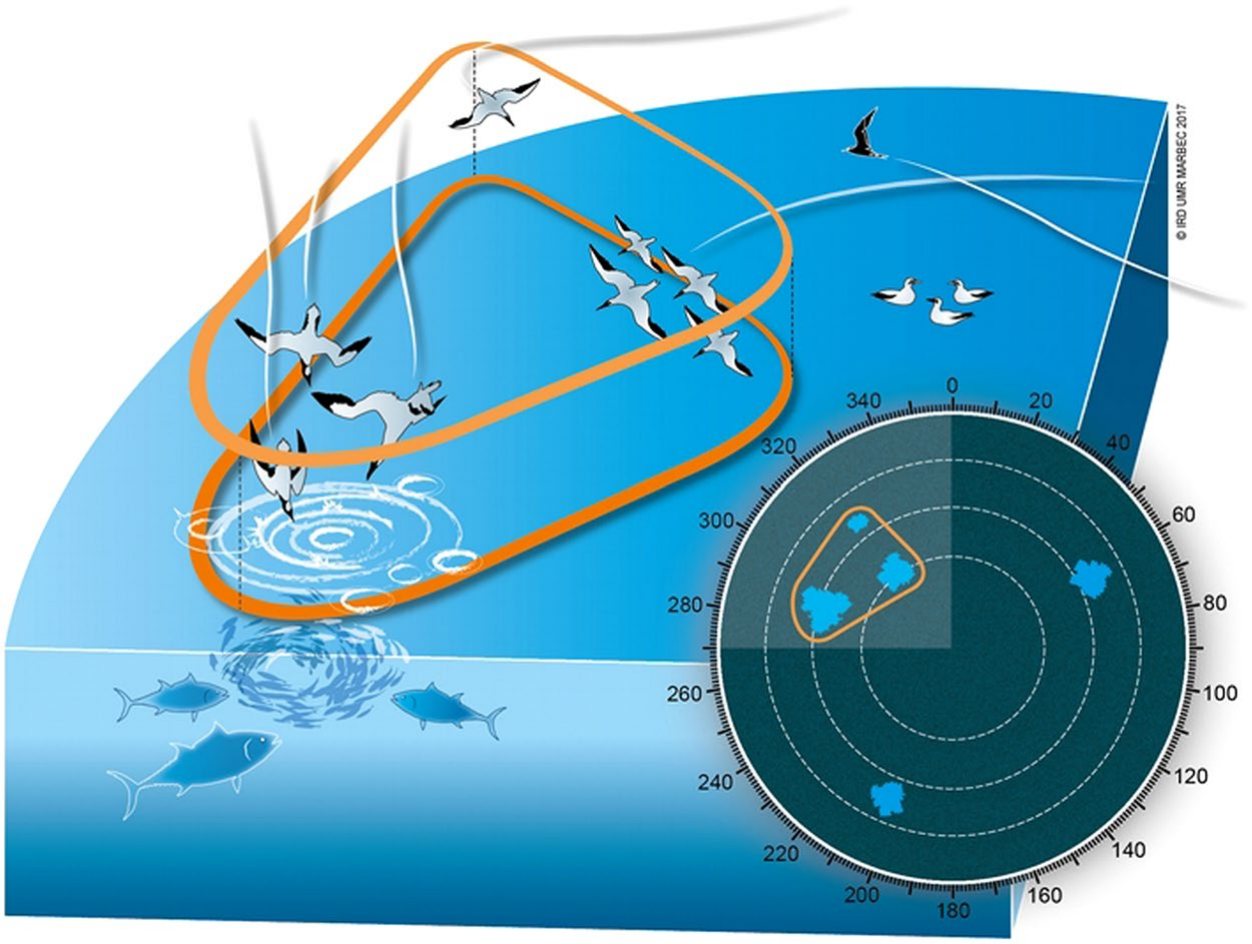
This illustration depicts an hypothetical oceanic scene with different seabirds and its possible appearance in the radar screen. The information relevant to this work are: (1) echoes in the radar can be either one bird or a group of birds foraging or transiting (referred to as “seabird-echoes” or “seabirds groups” in the text); (2) small seabirds and seabirds sitting on the water are unlikely to be detected by the radar; (3) when echoes are spaced such that they appear close together (see how in methods), they form a cluster of echoes (referred to as such in the text). Clusters might then be considered as “groups of seabirds groups”.

We recorded 196,053 radar screen images, 37,333 of which were selected based on their quality and setting characteristics (selected images came from 27 days out of 73, see methods). Seabird-echoes were observed in all images, and their numbers ranged from 1 to 292 per image (median: 34). Echoes could represent single individuals or groups of individuals (always in flight, Fig. 1), but we had no possibilities to distinguish them, nor to determine species.

**Figure 2:**
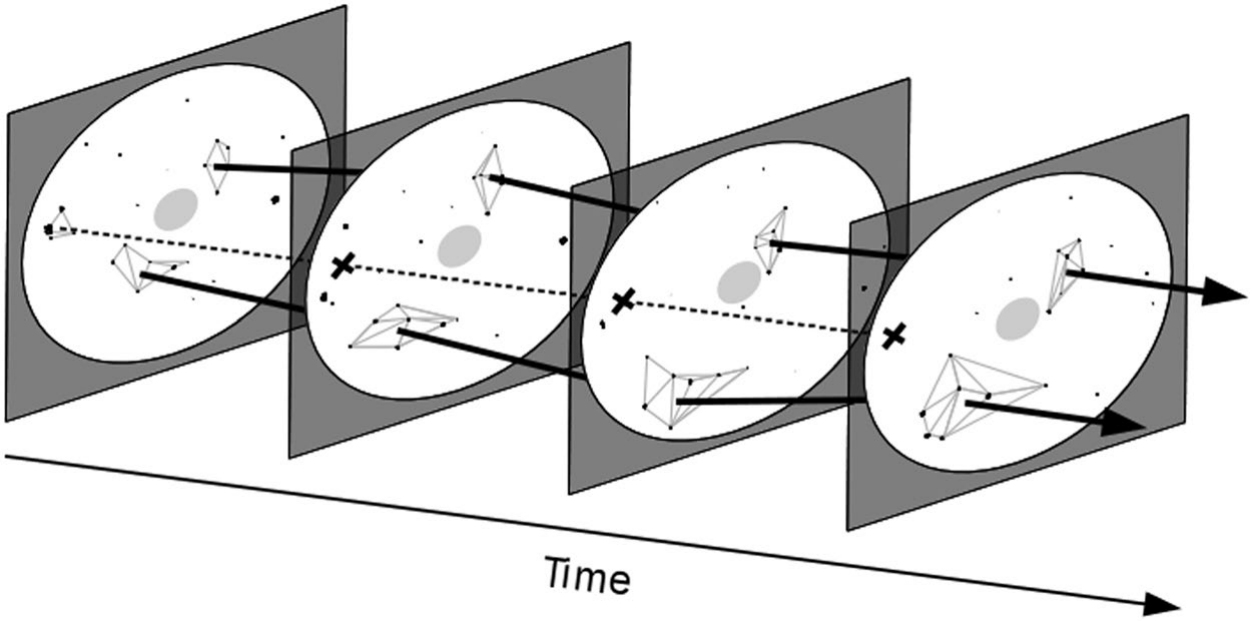
This illustration depicts the tracking procedure of clusters’ centroids. The four panels correspond to four successive schematised radar images. In each image, white circle represents the scanned radar disk, black dots are seabird-echoes, and the central light grey circle symbolises the central saturated zone where no information is available. Black arrows crossing radar disks link clusters of echoes showing spatial and temporal consistency. Dotted line and black crosses stand for missing linkages of a cluster’s centroid in three successive images, thus closing the tracking procedure for this cluster. The latter is invalidate by the tracking procedure and considered as a non-consistent structure in seabirds distribution.

The density-based spatial clustering of echoes (see methods) resulted in the determination of 123,357 clusters. These clusters indicate patterns of coherent density in echoes’ distribution, i.e aggregative structures of groups of seabirds (Fig. 1). Among these clusters, 80,639 (65.4%) were kept as they showed temporal consistency and allowed for the tracking of 7,657 individual clusters in time (Fig. 2). Clusters’ consistency in time is unlikely to happen at random and was used as a reliability criteria. Erratic clusters were not taken into account (Fig. 2).

9.9% of images did not contain any cluster. In these cases, seabirds distribution did not show any temporally nor spatially consistent pattern. One third (30.9%) contained a single cluster. Half of images (50.8%) contained 2 to 4 clusters. In the remaining 8.4% images, the number of clusters ranged from 5 to 22. The proportion of cluster-associated echoes in an image ranged from 0% to 100% (median: 63.9%). In 75% of images, more than 41.7% of echoes belonged to one or several clusters. At least one quarter of echoes are included in one or several clusters in 85% of images.

**Figure 3:**
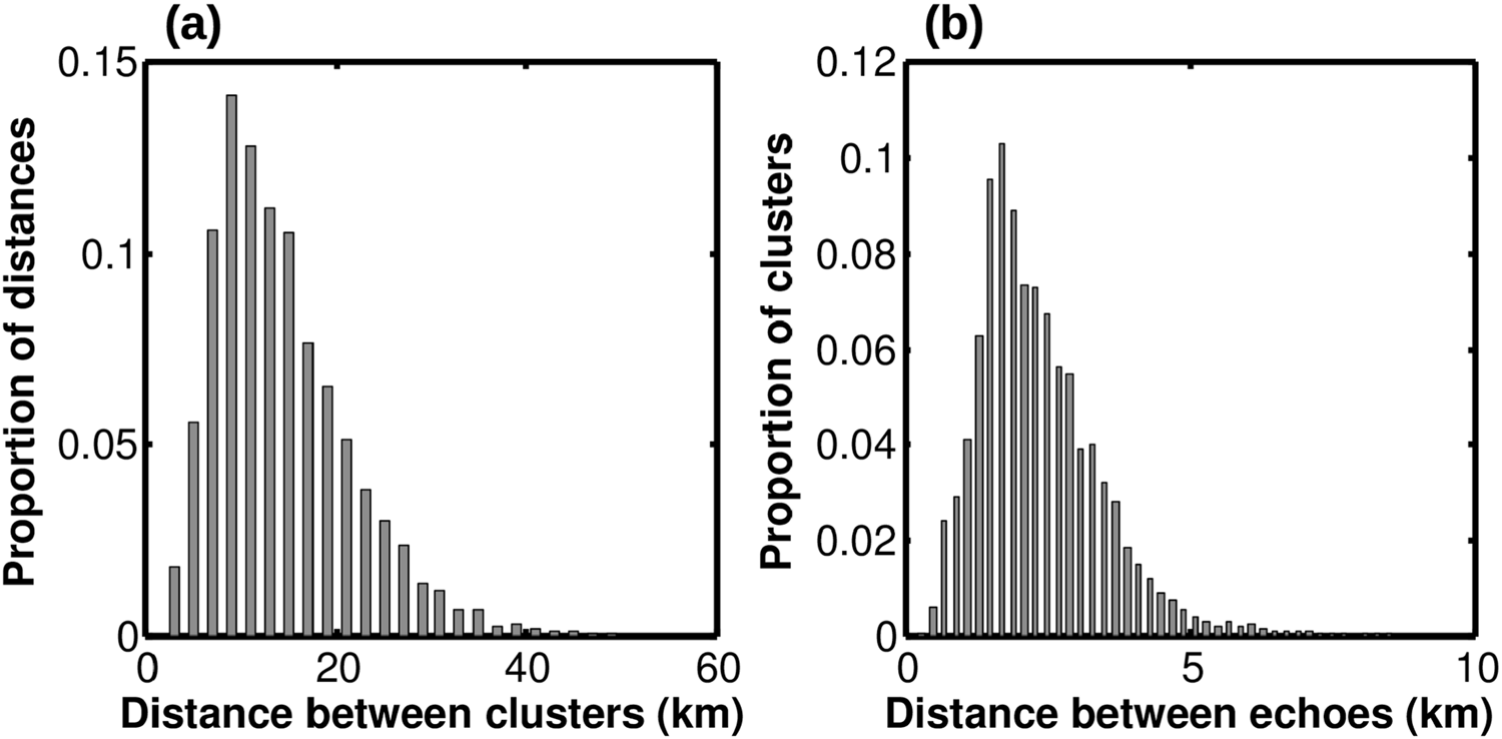
(**a**) Distribution of distances between synchronous clusters’ centroids (km). (**b**) Distribution of the mean distance between echoes within clusters (km).

Clusters’ centroids were 12.9 km apart (median), with minimum and maximum distances being 2.0 km and 50.8 km. Half of inter-clusters distances lied between 9.0 and 18.2 km and 95% were less than 27.8 km (Fig. 3a).

**Figure 4:**
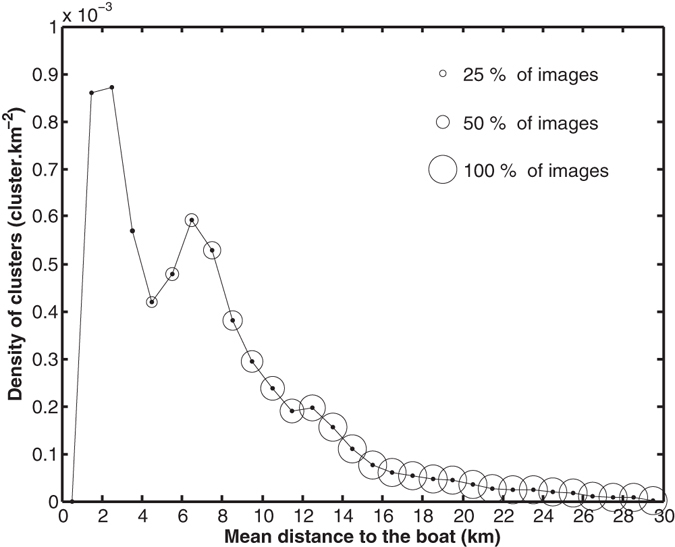
Density of clusters (cluster.km^−2^) as a function of the distance to the boat (km). Each cluster is located at its mean position. Circles indicate the proportion of images contributing to the estimates; this proportion is lesser at small distances due to numerous images with saturated and unexploited zone in the first kilometres from the boat.

Clusters’ centroids were found in all the radar disk, from 1.5 to 29.5 km from the boat (Fig. 4). Less than 8% of images contributed to the estimation of clusters’ density in the range 0–3 km. Highest densities were found in the range 1–8 km, informed by 0.4% to 58.4% of images. Beyond 10 km, density decreased steadily from 2.9 * 10^−4^ cluster.km^−2^ to 2.2 * 10^−6^ cluster.km^−2^. Within this range, more than 75% of images provided information on echoes distribution and contributed to the estimation.

Using a tree-based classification procedure with three clusters’ descriptors (maximum extent, area, and mean distance between echoes), we were not able to distinguish between significant classes of clusters. The centre of mass of the three descriptors was characterized by an area of 47.1 km^2^ (corresponding to an equivalent diameter of 7.7 km), a maximum extent of 10.4 km, and a mean distance between echoes of 2.4 km. 95% of clusters showed areas in the 0.02–208.9 km^2^ range, equivalent diameters between 0.2 and 16.3 km, maximum extents between 0.9 and 27.6 km, and mean distances between echoes between 0.4 and 5.7 km (Fig. 3b).

The means of coefficients of variation for these three variables along clusters’ tracks were significantly different (p-value = 0 for two-by-two variables ANOVA tests). The maximum extent of clusters was the most stable metric along clusters’ tracks (Table 1) with a mean coefficient of variation (CV) of 0.4, compared to the mean CV of the distances between echoes in clusters (0.5) and the CV of clusters’ area (0.8).

**Table 1:** Descriptive statistics of clusters. Delaunay distances between clusters’ centroids correspond to the length of edges obtained by Delaunay triangulation on synchronous clusters’ centroids.

Variable	Median	Mean	Min	Max	Mean of coefficients of variation	Sd. of coefficients of variation	Maximum possible given the radar range of 30 km
Delaunay distances between clusters’ centroids (km)	12.9	14.3	2.0	50.8	—	—	60 km
Clusters’ area (km^2^)	21.9	43.2	0.02	505.2	0.8	0.4	2,827 km^2^
Equivalent diameter of clusters (km)	4.8	5.8	0.2	24.5	0.4	0.2	60 km
Maximum extent of clusters (km)	9.2	10.6	0.9	39.5	0.4	0.2	60 km
Delaunay distances between echoes in clusters (km)	2.1	2.3	0.4	8.5	0.5	0.1	60 km
Distance to the boat at starting point (km)	12.0	13.0	0.3	29.5	—	—	30 km
Distance to the boat at ending point (km)	11.9	12.9	0.1	29.5	—	—	30 km
Translation of clusters’ centroids (km)	3.2	3.8	0.03	36.9	—	—	60 km
Duration of clusters (h:min:s)	00:03:30	00:05:09	00:00:30	01:20:04	—	—	—

Clusters’ areas, equivalent diameters, maximum extents and Delaunay distances between echoes were averaged for each cluster during its lifetime. Coefficients of variation of the four previous metrics (along clusters’ tracks) were calculated for each cluster. The mean and standard deviation of all coefficients of variation are reported for the four metrics (columns 6 and 7). Distances to the boat at starting and ending points refer to the distance of clusters’ centroids to the boat at the beginning and at the end of clusters’ tracks. Translations of clusters correspond to the distance between their starting and ending centroids’ locations.

Clusters lasted 03 min 30 s (median) and 95% of them lasted less than 14 min 30 s. The maximal duration of a cluster reached 01 h 20 min (Table 1).

95% of clusters moved less than 9.1 km (translation between starting and ending location) during their lifetime (median: 3.2 km, see Table 1). 8.4% of clusters’ translations lengths were superior to their maximum extent and to the threshold distance used in the tracking procedure (4 km). These clusters tended to get closer or more distant from the boat in equivalent proportions (54.9% and 45.1% respectively).

During clusters’ lifetime, the boat and the clusters’ centroids tend to get closer or more distant from each other in equivalent proportions (52.4% and 47.6% respectively). Distances of clusters’ centroids to the boat at the starting and ending points of their track got similar distributions (Kolmogorov-Smirnov test, p-value = 0.39). Half of starting and ending locations of clusters were found in the range 0–12 km from the boat (Table 1). 95% of ending points of cluster tracks, as well as 95% of starting points, were located at more than 5 km from the radar circle edge.

## Discussion

To our knowledge, this is the first study describing the instantaneous distribution of seabirds at sea using an on-board fishing boat radar. Vessel or plane based transect survey of seabird allows for the estimation of their averaged distribution in time but does not permit to record instantaneous seabirds distribution dynamically. In contrast, we were able to track every few seconds the distribution of a whole seabird community in an area larger than 2800 km^2^ (30 km radius).

Despite recent regain of interest in radar aeroecology^29^, the first description of bird-echoes in radar observations dates from the earliest days of radar use^30^. Since fishermen use radars to cue on the presence and behaviour of seabirds, and consequently tune both radar settings and display for this purpose, we are strongly confident that the echoes we selected were seabirds. The occurrence of other fishing vessels was rare given the remote location of the boat in the Indian Ocean, and easily filtered out from seabird-echoes (e.g. echoes from boats are larger, not as rounded, and move slower than echoes from seabirds). However, we could not assess whether one echo represented one or several birds. It is also likely that some isolated individuals have been missed due to their small size or relative orientation. Finally, we were also unable to know the species involved. Vessel-based visual countings^31^ documented that the most numerous species in the study zone (Comoros, Glorieuses and Seychelles archipelagos) were sooty terns (*Sterna fuscata*) and wedge-tailed shearwaters (*Puffinus pacificus*), which are oceanic foragers (in opposition to inshore foragers) that associate with tuna or marine mammals for feeding. As in our dataset, echoes are seen to split and merge, there is a great probability that they stand for groups of individuals of these two species. However, we do not exclude the potential detection of solitary and larger individuals such as frigatebirds, which are often found in association with diverse seabirds species and sub-surface predators for feeding^15^.

We showed that the seabirds distribution was generally structured in few clusters, i.e. homogeneous density patterns of echoes that were consistent in time. To our knowledge, these structures as well as their temporal and spatial scales have never been described before. Previous ship-transect observations evidenced some seabirds aggregations over ~4.7–23 km segments, the nature of which remained, however, enigmatic^26^. Moreover, the non-negligible proportion of clustered echoes within images as well as the number of synchronous clusters (one or more clusters in ~90% of images) indicate that such structures are frequent and involve a significant part of the in-flight seabirds community. More rarely, the number of clusters simultaneously observed could exceed 4 and reach 22. In a similar proportion among images, no clusters were observed. This variability suggests that the formation of clusters within the seabirds distribution is not the exclusive deployment strategy at sea.

Size-wise, both maximum extent and equivalent diameter of clusters (about 9–10 km and 5–6 km, respectively) were in the same order of magnitude as previous estimates of flock recruitment distances during chumming experiments ranging from 4.9 to 11.3 km^19^. Reaction distances observed in Cape gannets^8^, reached 21.1 km for a bird in flight responding to a raft of con-specifics, and almost 40 km for a flying bird reacting to a large flock (100–150 gannets). The coherence between maximum extents of clusters and the reaction distances estimated in these two papers suggests that groups of seabirds composing a cluster are all in sight of each other, and they can potentially all interact. Moreover, ranges of both maximal extent of clusters and their equivalent diameter (from <1 km to 40 and 25 km respectively) indicate diverse configurations of the seabirds distribution, allowing for interactions at small scale (1 km) as well as sub-meso-scale (10–40 km). In a foraging context, pelagic seabirds face unpredictable locations and abundances of prey patches, as well as variable numbers of seabirds groups at sea, as we saw in this study. This induces different needs for information^32^, and, as a consequence, different spatial distributions of seabirds. Ranges of spatial descriptors of clusters might then encompass different scales of information use among seabirds groups. In addition, the vast majority of synchronous clusters’ centroids are distant from 2.0 to 27.8 km from each other. Within this range, seabirds of different clusters were thus potentially able to detect changes in distant groups’ behaviour too. Considering seabirds groups as nodes and information transfers as links, a foraging network structure could potentially emerge in such seabirds groups distribution, similarly to the emergence of social contagion in fish schools^33^.

Clusters’ area was the most variable property of clusters along their tracks. This shows important changes in clusters’ structure as echoes appear or disappear (e.g. when seabirds groups split, merge or when seabirds sit on the water), as well as changes in their shapes (Fig. 2). Estimates of clusters’ speeds along their track were thus not computed because of the high variability of clusters’ centroids location resulting from added or lost echoes at clusters edges. Interestingly, distances between echoes within a cluster were less variable and in the order of 2.1 km. This distance might represent a comfortable distance at which seabirds can detect and interpret other groups of seabirds’ behaviours. The maximum extent of clusters was the less variable metric and in the order of 9.2 km. The relative stability of these two structural descriptors suppose an active control of inter-group distances.

Assuming the presence of a foraging network in which groups of seabirds maintain a visual contact with other groups to detect potential feeding activities, we could ask if a change in visibility conditions, which influences detection distances, impacts the geometry of clusters. Recordings associated to situations of bad weather were filtered out and could not inform us on the seabirds distribution. Furthermore, in our dataset, we only got a single recording of a “day-night-day” succession in radar images (April 8 to April 9, 2012; 5786 images). In this sequence, flying seabirds were detected both day and night. ANOVA tests were performed to inspect whether clusters’ metrics showed different statistical mean during day and night. Between day and night, significant differences were observed in the distributions of the number of clusters (increase of 17%, p-value = 10^−19^), the proportion of clustered echoes (increase of 7%, p-value = 3 * 10^−6^), the mean distance between echoes (decrease of 13%, p-value = 10^−5^) and the clusters’ duration (increase of 79%, p-value = 2 * 10^−16^). During night time, clusters thus involved a larger proportion of flying seabirds groups and lasted longer. Clusters were also denser and groups of seabirds more spatially clumped. In the context of information transfers between seabirds, both tendencies suggest longest interactions in low light conditions, allowed by groups of seabirds getting closer from each other. Interestingly, this night coincided with the full moon period, so seabirds might have relied on vision even at night, possibly backed-up with sound emissions^34^. However, this hypothesis emerges from a comparison involving only one night, and needs to be tested with more data.

There was no tendency for clusters to get closer to or away from the boat. Reversely, this suggests that the boat did not systematically navigated towards these structures. Clusters were either not visually detected by the fisherman on the radar screen, either not interpreted as being presumably linked to the presence of tuna. In addition, the few significant clusters’ displacements were not oriented in relation to the boat’s location. Centroids’ movements during clusters’ lifetime were within the range of the clusters’ equivalent diameter and lesser than clusters’ maximum extent. In most cases, the cluster’s area at its first appearance overlapped the cluster’s area at this last appearance. The vast majority of clusters thus does not show a significant translation during their observation. Consequently, they may be considered as virtually non-moving structures.

Despite no clear evidence of the boat on seabirds distribution could be credited, highest densities of clusters were found close to the boat (in a 1–8 km range). Given our observation protocol, we can not disentangle whether seabirds were reacting to the presence of the boat (attraction, avoidance) or the boat was reacting to seabirds presence and behaviour (fishing strategy). It is possible that the saturated zone around the boat (with a median radius of 7.1 km) might have precluded such observation. Indeed, seabirds can adopt a vessel-attendance (both vessel-attraction and boat-following) in the presence of fishing boats releasing offal^35^ or during ship survey^36^. A ship-generated stampede has also been described^37^ while a ship approached tuna or dolphin-associated seabird feeding flocks. Seabirds then sit on the water or remain in flight to wait for the prey to be accessible again. Both attraction to the boat and sitting on water are behaviours influencing the presence and distribution of echoes in radar images. Such interactions were not accessible to our observation if they occurred in the central saturated zone of the image i.e. at few kilometres from the boat. Seabirds can be attracted to fishing boats at distances of 7 km^14^ or even 30 km^38^, and show behavioural changes at distances 3.1 km^38^ up to 11 km^39^. Tuna purse-seiners such as the one we used for this experiment are not known to highly attract seabirds in comparison with longliners. Given the large detection distance of boats by seabirds, we could not fully exclude the possibility that the quantity of seabirds in our dataset was influenced by its presence. However, except in its vicinity, we see no reason for the boat to generate aggregative patterns in seabirds distribution at large distances and we are strongly confident that the seabirds distribution – i.e. aggregative patterns occurrence – was not generated by the boat presence. We need more data on seabirds’ occurrence and distribution in the first ten kilometres to the boat to address pertinently scales of potential boat’s influence, especially during different activities (fishing, cruising).

Ninety five percent of clusters lasted less than ~15 min and were therefore rather ephemeral. This property could be due to a partial observation of clusters as the boat navigates and may not scan the cluster during its whole lifetime, especially if the latter moves away from the boat. Still, we are confident that this is not the case in our study because (1) most clusters did not move significantly, and (2) clusters often vanished while still being in the radar disk, as very few clusters’ ending locations were found close to the radar disk edge.

There is growing evidence that top-predators, and especially seabirds, can be strongly associated with mesoscale to submesoscale oceanographic features (1–10 km) during days to weeks^40^. These features are mostly eddies^41^, filaments^42^, and fronts or ice edges^43–46^. Nonetheless, the temporal consistency of clusters was not in accordance with the relative stability of such oceanographic features, suggesting that clusters emerged from distinct behavioural processes. Our observations do not exclude that the spatial occurrences and dimensions of clusters might be concordant with meso-scale oceanographic features, while their temporal dimension might be rather linked to information exchanges between individuals or groups.

In addition to reacting to flying hetero-specifics^23^ and con-specifics^24^, seabirds are known to react to rafts of con-specifics^8^, as well as sub-surface predators and boats^14^. All of these can be used as cues of the presence of prey and so take part in the information network. Except boats, these cues are not visible on radar images. Our observation of the network components is then incomplete. Seabirds distribution was described through density-based clustering of echoes, i.e. in a “flying-seabirds” context only. Clusters sizes described in this work are thus to be considered as minima.

This work focused on describing a new level of distribution in seabirds populations. Although the results presented here do not prove its existence, they were compatible with the presence of a foraging network within seabirds and therefore do not invalidate such hypothesis. Future work will determine where and when these coherent structures occur in relation to the oceanographic context. Further, the possibility to analyse echoes’ tracks gives us the potential to understand fine-scale behavioural processes within clusters. We showed that radar from moving platform such as boats can be used to study seabirds, provided that weather conditions are favourable. Because radars can detect patterns invisible from a boat but may not detect smaller birds or inform on species, radar studies are complementary to transect surveys, and we encourage studies combining both information.

## Methods

### Data collection

Modern tuna purse-seiners are equipped with two radars, one dedicated to the navigation, and another one exclusively dedicated to seabirds observation. For the latter, fishermen tune both settings and display to best observe flying seabirds distribution and behaviour and assess the presence of tuna schools. During a scientific survey^47^ (western Indian Ocean, latitude [18°S–4°S], longitude [41°E–62°E], from March 28th, 2012 to August 8th 2012) on-board the tuna purse-seiner Torre Giulia, an external video-card (DVI2USB frame-grabber, Epiphan System Inc., Canada) was used to save images of the seabirds-dedicated radar screen every 2–40s (mostly 30s) onto a laptop computer. This radar (marine surveillance radar FAR-2137, S-Band, 30 kW, Furuno) scanned a circular area of 30 km radius around the boat. Images were saved as *.png files (Portable Network Graphic, i.e. lossless compression format) and analysed after the survey. On 73 days of recordings, the number of daily saved images ranged from 2 to 5879 (2766 images per day on average).

### Data processing

Beside radar echoes, images contained many information such as radar settings (range, orientation, rain and sea-clutter corrections, gain set) and boat situation (location, course, speed). These pieces of information were put in numerical format using an optical character recognition algorithm (OCR, adapted from ref. 48). Image analysis routines were used to select echoes (see paragraphs 1. and 1.1. below).

Echoes’ centroid relative location in the image, area (in pixels), and maximum intensity (color codes from 1 to 30) were identified. Knowing the true location of the boat, the relative location of the echoes in the image, and the range and orientation of the radar image, we calculated the echoes’ longitudes and latitudes. Date and hour of each image were obtained from its recording time. Before any identified echo could be used in the study, preliminary filters were applied:

1. Depending on the radar settings and the weather, echoes can be a number of different things, such as waves, rainfalls, boats and seabirds in flight. Rainfalls appear typically as very large, strong and coherent echoes whereas waves appeared as small, weak and incoherent echoes all over the disk scanned by the radar. The radar settings were chosen by the captain of the boat so that seabirds are best visible given the weather situations. This was usually obtained with a relatively large gain value (generally levels 70 to 76 out of 100 levels). Considering the radar range of 30 km, echoes assimilated to flying seabirds (referred to as “seabird-echoes”) probably mainly stood for groups of seabirds, and rarely for individual and large seabirds (Fig. 1). This was confirmed by common observations of seabird-echoes that were seen splitting and merging.
  The human eyes are very efficient in distinguishing these various types of echoes, and more generally in detecting signal from noise. We used this ability to manually select thresholds of intensity and area to numerically filter out unwanted echoes, as suggested in ref. 49. Consequently, selected echoes had a maximum reflection intensity of at least level 18 (out of 30 levels) and areas of less than 250 pixels.
  Seabird-echoes were clearly distinguishable from noise-echoes due to their temporal and spatial consistency along successive images. We used this property to classify previously selected echoes into a class of seabird-echoes and a class of remaining noise. The latter originated from clutter on edges of rainfall-echoes and was hard to isolate from seabird-echoes on area and intensity-based criteria only. Consequently, we adapted the tracking algorithm “simpletracker”^50^ so as to include four tracking constraints, compatible with potential tracks obtained from seabirds. The algorithm was constraint by: (i) a maximum linking speed of 80 km between echoes’ centroids of successive images; (ii) a maximum gap closing (maximum number of images in which an echo might temporarily disappear) of 3, which corresponds on average to 1 min; (iii) a maximum azimuth difference of 90° between tracks of two successive images; (iv) a maximum azimuth difference of 30° between tracks of two images separated by 1 to 3 images. All echoes that were not members of tracks of at least 5 points (i.e appearing during 5 images or 2 minutes), were considered too inconsistent to be reliably seabird-echoes and thus classified as noise. After this, selected echoes were structurally (area, intensity), spatially and temporally (long tracks) consistent with echoes of seabird groups as we could identify them visually in radar images.
2. At distances very close to the radar, a saturated zone typically occurs at the centre of the radar disk. No valid echoes can be determined within this distance (which can be several kilometres) and this large artefactual echo was therefore filtered out after an image closing (dilatation/erosion) procedure.
3. In some instances, the proximity of several echoes led their pixels to appear contiguous on the screen even though the reflective intensities clearly showed distinct echoes. Several echoes could then be artificially lumped together and appear as one larger echo in the image. This artefact was overcome using a watershed transform algorithm^51^. The lattice of intensity values of pixels forms a landscape of peaks and valleys on the 2D plane, and the watershed aims at finding dividing lines between valleys and hence, in our case, between contiguous echoes. By replacing dividing lines pixels by a zero in reflection intensity, we separated contiguous echoes.
4. Images recorded while the radar was in stand-by mode, or images containing huge echoes of rain or various artefacts were deleted as they could not accurately describe seabirds distribution. Thus, images were valid if the number of saturated pixels (central saturated area + rains + valid echoes) did not exceed 10% of the radar circle area, and if the radius of the central saturated disk did not exceed 15 km (half of the radar disk range).

### Data analysis

The observation of flying seabirds groups by radar is biased by two characteristics of the radar physics itself. First, the signal is attenuated when the distance to the boat increases. This is automatically corrected by signal processing inside the radar box before visualization on the screen. Second, radar pulse beams expand radially from the source, thus covering more space at larger distance than at close distance. Consequently, the resolution of the detection decreases with the distance to the radar. Thus, the number of detected echoes theoretically reduces with the distance to the boat, and/or some attenuation of seabirds signal remains.

The loss of echoes at large distance to the boat is also affected by the gain (the larger the gain, the lesser the slope). In order to avoid extreme signal attenuation or noise due to temporary changes of gain values, we selected images whose gain value was part of the seven most represented ones, i.e values in [70, 76] from the range [0, 100] and used a clustering method known for its robustness against density fluctuations.

### Adaptive spatial clustering based on Delaunay triangulation

Echoes distribution was described by an Adaptive Spatial Clustering based on Delaunay Triangulation (ASCDT)^52^. This density-based clustering is a non parametric method where clusters identification is led by density homogeneities within a set of points. This method can identify clusters of arbitrary shapes, with different densities, and avoid noise effects on clusters connection. The specificity of ASCDT algorithm^52^ is the use of a two-steps strategy to detect clusters in a spatial set of points. Two parameters have to be chosen (a global cut-off value A and a local cut-off value B for the edges of the triangulation) to set the sensitivity of the algorithm to density variations of points in space, and thus defining clusters.

To choose the two cut-off values A (global scale) and B (local scale), we sampled 100 representative images from the dataset. The gain values and the number of seabird-echoes of these images spanned the corresponding ranges found in the dataset. The ASCDT algorithm was processed on the echoes’ centroids positions in each image. We tested 278 combinations of A and B values ranging from 0 to 5. For each combination and each image, we saved the number of clusters obtained, as well as the proportion of clustered echoes over the total number of echoes in the image. By construction, the smaller A and B values the smaller the proportion of clustered echoes. We selected ranges for A and B where the number of clusters and the proportion of clustered echoes were the less sensitive to A and B changes. We avoided extreme A and B values for which clustering systematically led to the detection of a unique cluster of all points in the image or to the absence of cluster. The choice of A and B, within their ranges of low sensitivity (0.25 to 1.5), was concluded by visual inspection of results. This process led to set parameters A and B to 0.5 and 0.75 respectively.

Clusters were finally validated if they contained at least 4 echoes and if they showed temporal consistency (Fig. 2). As a matter of fact, erratic clusters were not expected to be consistent from one image to the next. We thus used a tracking algorithm (simpletracker)^50^ on clusters’ centroids. The algorithm was constraint by: (i) a maximum linking distance of 4 km between centroids of successive images; (ii) a maximum gap closing (maximum number of images in which a cluster might temporarily disappear) of 3, which corresponds on average to 1 min. All clusters that were not members of tracks of at least 4 points (i.e appearing on 4 images), were considered as inconsistent (i.e. not reliably measured) and were not used further for the description of the seabirds’ groups distribution (Fig. 2). These clusters were not geometrically wrong, but rather, they were detected too weakly to be considered as representative structures.

### Clusters metrics

For each validated cluster we computed its contour (convex hull) and corresponding area (in number of pixels) from the position (pixels indices) of echoes composing it. Knowing the radar disk range (in km) and the radar disk radius (in pixel), we could calculate the area of one pixel (3.7 * 10^−3^ km^2^) and then convert each cluster’s area in km^2^.

Each cluster centroid’s position and centroid’s distance to the boat were also calculated in each image. From the clusters’ tracks, supplementary metrics were computed: the mean location of clusters’ centroids, their location at the beginning and at the end of the track (referred to as “starting point” and “ending point”), the duration or “lifetime” of clusters deduced from track length (presence during n successive images; Fig. 2) and time step between images, and the number of synchronous clusters found in each image. The distance between synchronous clusters’ centroids was calculated on 1495 images separated by more than 6 min (quantile 0.75 of clusters’ duration) to minimize autocorrelation. Distances between synchronous clusters correspond to the edges’ lengths of a Delaunay triangulation on clusters’ centroids in the same image. The inter-clusters distances’ distribution was obtained from 6 919 edges lengths.

Structural metrics were averaged along each cluster tracks. The equivalent diameter of a cluster was deduced from its area, using the equation: $Equivalent\,Diameter=2\times \sqrt{Area/pi}$. The number of echoes composing the cluster was saved as well. From positions (latitude, longitude) of seabird-echoes in each cluster, we deduced the maximum distance between two echoes within the same cluster (referred to as the “maximum extent” of a cluster), the mean distance between echoes of a cluster, and the coefficient of variation of these metrics (standard deviation/mean).

As the sequence of images was not regular in time and could be empty for minutes or days, we defined different sequences in the dataset as groups of images being separated by more than 1 min 30 s. This threshold corresponds to the maximum gap closing used in the tracking procedure of clusters. Consequently, clusters’ tracks beginning in the first image of a sequence, or ending in the last image of a sequence, were potentially artificially truncated. Structural and temporal clusters’ metrics were therefore computed only using clusters that were not artificially truncated, whereas all the clusters contributed to the estimate of the number of clusters per image.

## Electronic supplementary material

Example of a one-hour recording of seabird-echoes in radar images
